# Carotid chemoreflex and muscle metaboreflex interact to the regulation of ventilation in patients with heart failure with reduced ejection fraction

**DOI:** 10.14814/phy2.14361

**Published:** 2020-02-05

**Authors:** Alessandro C. Machado, Lauro C. Vianna, Erika A. C. Gomes, Jose A. C. Teixeira, Mario L. Ribeiro, Humberto Villacorta, Antonio C. L. Nobrega, Bruno M. Silva

**Affiliations:** ^1^ Laboratory of Exercise Sciences Department of Physiology and Pharmacology Fluminense Federal University Niterói RJ Brazil; ^2^ Latin American Institute of Life and Nature Sciences Federal University of Latin American Integration Foz do Iguaçu PR Brazil; ^3^ Faculty of Physical Education University of Brasília Brasilia DF Brazil; ^4^ Antonio Pedro University Hospital Faculty of Medicine Fluminense Federal University Niterói RJ Brazil; ^5^ Department of Physiology Federal University of São Paulo São Paulo SP Brazil

**Keywords:** breathing, circulatory occlusion, exercise, heart disease, hyperoxia

## Abstract

Synergism among reflexes probably contributes to exercise hyperventilation in patients with heart failure with reduced ejection fraction (HFrEF). Thus, we investigated whether the carotid chemoreflex and the muscle metaboreflex interact to the regulation of ventilation (${\dot{V}}_{E}$) in HFrEF. Ten patients accomplished 4‐min cycling at 60% peak workload and then recovered for 2 min under either: (a) 21% O_2_ inhalation (tonic carotid chemoreflex activity) with legs’ circulation free (inactive muscle metaboreflex); (b) 100% O_2_ inhalation (suppressed carotid chemoreflex activity) with legs’ circulation occluded (muscle metaboreflex activation); (c) 21% O_2_ inhalation (tonic carotid chemoreflex activity) with legs’ circulation occluded (muscle metaboreflex activation); or (d) 100% O_2_ inhalation (suppressed carotid chemoreflex activity) with legs’ circulation free (inactive muscle metaboreflex) as control. ${\dot{V}}_{E}$, tidal volume (V_T_) and respiratory frequency (*f*_R_) were similar between each separated reflex (protocols a and b) and control (protocol d). Calculated sum of separated reflexes effects was similar to control. Oppositely, ${\dot{V}}_{E}$ (mean ± *SEM*: Δ vs. control = 2.46 ± 1.07 L/min, *p* = .05) and *f*_R_ (Δ = 2.47 ± 0.77 cycles/min, *p* = .02) increased versus control when both reflexes were simultaneously active (protocol c). Therefore, the carotid chemoreflex and the muscle metaboreflex interacted to ${\dot{V}}_{E}$ regulation in a *f*_R_‐dependent manner in patients with HFrEF. If this interaction operates during exercise, it can have some contribution to the HFrEF exercise hyperventilation.

## Graphical abstract

Our results support that the carotid chemoreflex and the muscle metaboreflex interact to the regulation of ventilation in patients with chronic heart failure with reduced ejection fraction (HFrEF), which may have implication for exercise hyperventilation in this population. Furthermore, the effect mediated by separated reflexes was not associated with the effect yielded when both reflexes were simultaneously active. Hence, reflexes interaction should be further explored to advance the understanding of exercise hyperventilation in HFrEF.

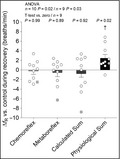

## 1 INTRODUCTION

Patients with heart failure with reduced ejection fraction (HFrEF) typically present with excessive ventilation (${\dot{V}}_{E}$) during exercise, for a given amount of carbon dioxide output ($\dot{V}$CO_2_) (Chua et al., 1997; Smith & Olson, 2019; Sullivan, Higginbotham, & Cobb, 1988; Van Iterson, Johnson, Borlaug, & Olson, 2017). This phenomenon is of clinical relevance, as the ${\dot{V}}_{E}$/$\dot{V}$CO_2_ ratio and the ${\dot{V}}_{E}$/$\dot{V}$CO_2_ slope are independent predictors of morbidity and mortality in patients with HFrEF (Arena, Myers, Aslam, Varughese, & Peberdy, 2004; Chua et al., 1997). The excessive exertional ${\dot{V}}_{E}$ in HFrEF is dependent on an increase of the respiratory frequency (*f*_R_) (Smith & Olson, 2019; Van Iterson et al., 2017) and it seems to be mostly mediated by enhanced drive to breathe (Tasoulis et al., 2014; Van Iterson et al., 2017). Multiple abnormalities in feedback mechanisms have been postulated to increase the central respiratory drive during exercise in HFrEF, including enhancement of afferent signaling from the central circulation (Taylor, Smetana, Frantz, & Johnson, 2015), lungs (Van Iterson et al., 2017), skeletal muscles (Olson, Joyner, Eisenach, Curry, & Johnson, 2014; Smith, Williams, Mitchell, Mammen, & Garry, 2005), carotid chemoreceptors (Clement, Pandit, Bascom, & Robbins, 1996; Morosin et al., 2018), and central chemoreceptors (Narkiewicz, Pesek, Van De Borne, Kato, & Somers, 1999). However, none of these reflexes, when separately activated at physiological levels, yields a ${\dot{V}}_{E}$ response close to 50% of the one observed during exercise (Forster, Haouzi, & Dempsey, 2012; Parkes, 2017). Therefore, interaction among possible underlying neural mechanisms perhaps plays an important role for the regulation of ${\dot{V}}_{E}$ and respiratory pattern during exercise in HFrEF.

Patients with HFrEF may present with increased carotid chemoreceptors activity under normoxic rest (Despas et al., 2012; Ponikowski et al., 1997), most likely due to low carotid bodies perfusion (Ding, Li, & Schultz, 2011). In addition, carotid chemoreceptors may be further activated by neural (Edgell & Stickland, 2014; Silva et al., 2018) and humoral (Linton & Band, 1985; Pandit, Bergstrom, Frankel, & Robbins, 1994) signals derived from skeletal muscle contractions. Thereby, the contribution of the carotid chemoreflex to cardiorespiratory regulation seems to enhance during exercise (Niewiński et al., 2013; Stickland, Miller, Smith, & Dempsey, 2007). In contrast, however, the carotid chemoreflex does not seem to contribute to ${\dot{V}}_{E}$ and respiratory pattern regulation during recovery from exercise in humans (Clement et al., 1996; Paula‐Ribeiro et al., 2019). Thus, an interaction among the carotid chemoreflex and other reflexes that operate during exercise, but not during recovery, possibly exist (Edgell & Stickland, 2014; Gujic et al., 2007; Scott et al., 2000).

An interaction among signals derived from metabolically sensitive thin‐fiber muscle afferents and other signals present during exercise may also exist. Inhibition of sensitive thin‐fiber muscle afferents via intrathecal fentanyl infusion at the lumbar level reduced ${\dot{V}}_{E}$ and *f*_R_, but did not change tidal volume (V_T_) during moderate to heavy‐intensity exercise (65% peak power) in patients with HFrEF (Olson et al., 2014). This evidence consequently suggests that the muscle metaboreflex plays a role for the ${\dot{V}}_{E}$ and *f*_R_ regulation during exercise in HFrEF. On the other hand, it is long known that muscle metaboreflex activation at rest, via postexercise circulation occlusion, commonly fails to maintain ${\dot{V}}_{E}$ and *f*_R_ above an unconcluded control condition in patients with HFrEF (Olson, Joyner, & Johnson, 2010; Scott, Davies, Coats, & Piepoli, 2002; Scott et al., 2000). One possible explanation for such divergent findings is that the muscle metaboreflex drives the ${\dot{V}}_{E}$ and respiratory pattern only when it interacts with other signals that operate during exercise. In support, a recent study revealed metabolites arrest at rest did not affect ${\dot{V}}_{E}$ and respiratory pattern (Lam, Greenhough, Nazari, White, & Bruce, 2019). However, when the same procedure was employed during exercise, it provoked ${\dot{V}}_{E}$ increase in a *f*_R_‐dependent manner in healthy young adults (Lam et al., 2019).

Although the separated effect of the carotid chemoreflex (Chua, Ponikowski, Harrington, Chambers, & Coats, 1996; Edgell et al., 2015) and the muscle metaboreflex (Olson et al., 2010; Scott et al., 2002, 2000) to the control of ${\dot{V}}_{E}$ and respiratory pattern has been previously studied in patients with HFrEF, the interaction between these reflexes remains uninvestigated. Noteworthy, the carotid chemoreflex and the muscle metaboreflex may interact at the level of the central nervous system and at the periphery. Afferent neurons from carotid chemoreceptors (Accorsi‐Mendonça, Castania, Bonagamba, Machado, & Leao, 2011) and metabolically sensitive thin‐fiber muscle afferents (Potts et al., 2003) synapse at close regions in the nucleus of the tractus solitarius where signals could be potentiated (Potts et al., 2003). Furthermore, activation of the muscle metaboreflex may increase carotid chemoreceptors activity via increased sympathetic nerve activity to the carotid bodies (O'regan, 1981). Therefore, the aim of this study was to investigate whether the carotid chemoreflex and the muscle metaboreflex interact to the regulation of ${\dot{V}}_{E}$ and respiratory pattern in patients with HFrEF. We hypothesized that simultaneous activation of these reflexes (i.e., “experimental sum”) would provoke greater ${\dot{V}}_{E}$ and respiratory pattern changes than the sum of separated reflexes changes (i.e., “calculated sum”), which consequently would indicate that the reflexes under investigation interact in a hyperadditive (i.e., synergistic or multiplicative) fashion to the regulation of ${\dot{V}}_{E}$ and respiratory pattern.

## 2 METHODS

### 2.1 Subjects

Ten patients (7 men) with HFrEF met the inclusion criteria and volunteered to participate in the study. Eligibility criteria for all were: age between 40 and 70 years, reduced left ventricle ejection fraction (Simpson < 45%), optimal pharmacological treatment, New York Heart Association (NYHA) class II or III, no hospitalization in the last 6 months, body mass index lower than 35 kg/m^2^, no chronic renal disease (creatinine > 1.5 mg/dl), no diabetes (fasting glucose > 126 mg/dl), no current smoking, no spirometric evidence of obstructive disease, forced expiratory volume in 1 s by forced vital capacity greater than 70% (FEV_1_/FVC > 70%) and resting oxygen saturation greater than 94%. Subjects were assessed at the Antonio Pedro University Hospital and at the Biomedical Institute of the Fluminense Federal University, in Niteroi, Rio de Janeiro State, Brazil. The Ethics Committee of the Fluminense Federal University approved the study (CEP‐CCM/HUAP 36681414.0.0000.5243), and the study conformed to the standards set by the Declaration of Helsinki. All subjects provided written informed consent to participate in the study.

### 2.2 Experimental protocol

The experimental protocol encompassed five visits. On visit 1, subjects underwent an incremental workload exercise. On visit 2, subjects were familiarized with cuffs inflation and constant workload exercise. In some cases, the familiarization visit was repeated to guarantee subjects’ adaptation with the protocol. Then, on visits 3 and 4, subjects underwent four constant workload exercise trials. Two trials were conducted per day, with at least 30 min of interval. At last, on visit 5, subjects’ carotid chemoreflex sensitivity was assessed. Subjects were asked to avoid strenuous physical activity and refrain from consuming caffeine for 24 hr before arriving for all visits. All visits were separated by at least 24 hr.

### 2.3 Experimental procedures

#### 2.3.1 Incremental workload exercise

Incremental workload exercise was performed on an electromagnetic cycle ergometer (ERGO‐FIT 167, ERGO‐FIT). Workload was progressively increased until volitional fatigue or until pedal cadence (60 rpm) could not be maintained despite strong verbal encouragement (Mezzani et al., 2009). Cardiac electrical activity (electrocardiogram) was continuously monitored for clinical purposes and arterial pressure (auscultatory method) was measured every 2 min.

#### 2.3.2 Constant workload exercise

Constant workload exercise was performed on an electromagnetic cycle ergometer at 60 rpm (ERGO‐FIT 167, ERGO‐FIT). Exercise intensity was set at 60% peak workload attained in the incremental workload exercise (Olson et al., 2010). Each constant workload exercise trial lasted 4 min. After that, subjects recovered seated on the cycle ergometer for 2 min. The use of two different gases (21% and 100% of O_2_ in the inspired air; i.e., normoxia and hyperoxia, respectively) and two circulatory states in the lower limbs (circulatory occlusion and free flow) were combined during the recovery period. A hundred percent of O_2_ was used in the inspired air in an attempt to provoke a fast and robust suppression of the tonic carotid chemoreceptors activity (Eyzaguirre & Lewin, 1961), whereas normoxia preserved tonic carotid chemoreceptors activity (Eyzaguirre & Lewin, 1961). Circulatory occlusion aimed to retain byproducts of skeletal muscle contractions within the lower limbs maintaining the activation of the muscle metaboreflex during the recovery period (Alam & Smirk, 1937). Free flow, in opposition, allowed removal of byproducts from skeletal muscle contractions, which should inactivate the muscle metaboreflex (Alam & Smirk, 1937).

**Figure 1:**
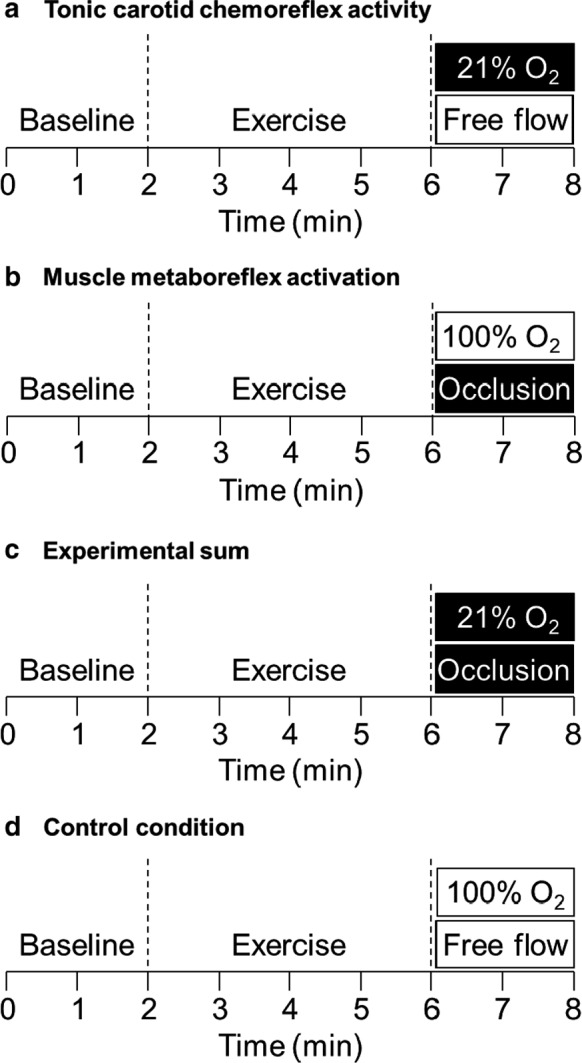
Illustration of experimental conditions. Conditions: (a) 21% O_2_ inhalation (tonic carotid chemoreflex activity) with free flow to the lower limbs (inactive muscle metaboreflex); (b) 100 O_2_ inhalation (suppressed carotid chemoreflex activity) with circulatory occlusion in the lower limbs (muscle metaboreflex activation); (c) 21% O_2_ inhalation (tonic carotid chemoreflex activity) with circulatory occlusion in the lower limbs (muscle metaboreflex activation); and (d) 100% O_2_ inhalation (suppressed carotid chemoreflex activity) with free flow to the lower limbs (inactive muscle metaboreflex)

In sum, four different conditions were formed (Figure 1): (a) 21% O_2_ inhalation with free flow to the lower limbs to maintain the tonic carotid chemoreflex activity without muscle metaboreflex activation; (b) 100% O_2_ inhalation with circulatory occlusion in the lower limbs to suppress the tonic carotid chemoreflex activity with muscle metaboreflex activation; (c) 21% O_2_ inhalation with circulatory occlusion in the lower limbs to have both reflexes simultaneously active; and (d) 100% O_2_ inhalation with free flow to the lower limbs to suppress the tonic carotid chemoreflex activity without muscle metaboreflex activation, which consequently was used as a control condition. The order of the first condition of a day was selected by randomization, and the opposite condition was used next. For example, if 21% O_2_ plus free flow was the first sorted condition, it had to be followed by 100% O_2_ plus circulatory occlusion in the same visit. In the next experimental visit, for the same subject, another randomization was done to select the order of the remaining conditions.

A breathing circuit was connected to the pneumotachometer at onset of the recovery period allowing either delivery of fresh air (21% O_2_ or 100% O_2_) or rebreathing of expired air (Banzett, Garcia, & Moosavi, 2000). We changed the level of O_2_ only during recovery due to two reasons: (a) to avoid a change in the O_2_ delivery to skeletal muscles during exercise, which could influence the metabolic response to exercise (Chua et al., 1996); (b) to avoid more than 2 min of exposure to hyperoxia, since this could lead to a paradoxical excitatory effect on the ${\dot{V}}_{E}$ regulation (Dean, Mulkey, Henderson, Potter, & Putnam, 2004). Subjects were blinded to the O_2_ concentration in the inhaled air. Rebreathing of expired air was used in an attempt to maintain the end tidal partial pressure of CO_2_ (P_ET_CO_2_) at the end‐exercise level, because circulation occlusion traps a large amount of CO_2_ in the lower limbs. As a result, the circulating level of CO_2_ reduces, which may depress carotid and central chemoreceptors (Olson et al., 2010). The target P_ET_CO_2_ for a given subject was the mean value observed during the last 20 s of the constant workload exercise conducted on the familiarization visit. A trained researcher (A.C.M.) manually controlled the amount of fresh gas delivered to achieve the target P_ET_CO_2_. So that, high P_ET_CO_2_ prompted increase of fresh air delivery consequently decreasing rebreathing, whereas low P_ET_CO_2_ prompted decrease of fresh air delivery consequently increasing rebreathing.

Circulatory occlusion at the level of the upper thighs (SC12^TM^, Hokanson) was initiated 5 s before the end of exercise through rapid cuff inflation (~0.5 s; E20, Hokanson). One cuff was used per limb. Cuffs were inflated simultaneously to 20 mmHg above peak systolic arterial pressure (SAP) recorded in the arm during the incremental workload exercise (Olson et al., 2010). Subjects were carefully familiarized with the circulatory occlusion on a day before the experiments. The familiarization started with cuff inflation at a pressure close to 100 mmHg at rest for about 30 s. Next, inflation pressure was increased and maintained for a progressively longer period. The increment of pressure and inflation duration was done according to the subjects’ tolerance, until the target pressure and duration was achieved at rest. Then, subjects performed the 4‐min constant workload exercise at 60% peak workload. Cuffs were inflated at the target level before the end of exercise and kept inflated for 2 min. The postexercise circulatory occlusion was repeated during the familiarization, in case perceived discomfort was greater than 3 on Borg's 0 to 10 scale or subjects held their breath while cuffs were inflated.

An oro‐nasal silicone mask (V2™, Hans Rudolph) was tightly adjusted to the subjects’ face to avoid air leak. A bidirectional pneumotachometer was connected to the mask (Prevent, MedGraphics) to allow the measurement of ${\dot{V}}_{E}$ and pulmonary gas exchange on a breath‐by‐breath basis (Ultima CPX^TM,^ MedGraphics). Cardiac electrical activity was measured by a 12‐lead electrocardiogram for clinical purposes (Welch Allyn CardioPerfect Workstation, Welch Allyn). Arterial pressure was measured via the auscultatory method.

#### 2.3.3 Carotid chemoreflex sensitivity

Carotid chemoreflex sensitivity was assessed via the transient hypoxia method (Chua & Coats, 1995). Subjects seated in the upright position on a comfortable chair. An oro‐nasal silicone mask (V2™, Hans Rudolph) was carefully adjusted to the subjects’ face. One side of a bidirectional pneumotach (Prevent, MedGraphics) was connected to the mask. Another side was connected to a piece composed of an one‐way valve and a stopcock. The one‐way valve avoided rebreathing of expired air. The stopcock allowed switch of inspired air between room air and pure nitrogen. The test began with a quiet undisturbed resting period of 5–10 min for measurement of baseline ${\dot{V}}_{E}$ and pulse oxygen saturation (SpO_2_). Then, subjects inhaled pure nitrogen for two to eight consecutive respiratory cycles. Inhalation of pure nitrogen was repeated six to 10 times. Repetitions were done after ${\dot{V}}_{E}$ and SpO_2_ returned to the baseline level, which usually took 2–5 min. SpO_2_ was measured at a hand finger (DRE Vida Multi‐parameter Monitor, Avante). Air flow and pulmonary gas exchange were acquired breath‐by‐breath with a metabolic analyzer (Ultima CPX^TM,^ MedGraphics). The average of the two highest consecutive ${\dot{V}}_{E}$ values, either during or after each nitrogen inhalation, was plotted against the respective lowest SpO_2_. Then, a linear regression was performed. The resulting slope was considered as an index of carotid chemoreflex sensitivity of an individual. Values up to 0.675 L/min/% were considered within the normal range (Chua et al., 1997).

### 2.4 Statistical analysis

Breath‐by‐breath respiratory data from 120 s of resting, last 20 s of exercise and 20‐s periods along recovery were averaged within each condition. In addition, data from 0 to 120 s of recovery were combined as a single average per condition per subject, and then, changes versus control were calculated. Sum of separated reflexes’ effects was named “calculated sum” and consisted of the sum of carotid chemoreflex change versus control and muscle metaboreflex change versus control [i.e., (protocol a − protocol d) + (protocol b − protocol d)]. “Experimental sum” was considered as the change provoked by simultaneous reflexes activities versus control (i.e., protocol c − protocol d). Changes versus control were considered the main study's endpoints. Thus, any change data located beyond 1.5 times the interquartile range for a variable, under a given condition, was considered as outlier. Raw data were contrasted to control via two‐way ANOVA, without outlier data. Changes versus control were compared among four conditions via one‐way ANOVA, both with and without outlier data. Fisher's post hoc was used after ANOVA, when needed. Changes versus control were contrasted to zero via single mean Student's *t*‐test. Associations were assessed via Pearson's correlation. The sample size for each analysis is reported, given that some respiratory data were not recorded due to technical problems with the metabolic analyzer and some outlier data had to be removed from inferential analyses. Subjects’ characteristics are reported as mean, standard deviation (*SD*), minimal (min) and maximal (max). Data compared by inferential statistics are presented as mean ± standard error of the mean (*SEM*). All inferential analyses were two‐tailed. Absolute *p*‐values were reported whenever possible. *p*‐values were interpreted taking into account the .05 value as an orientation, but not as cutoff, according to accumulating statisticians’ recommendations (Amrhein, Greenland, & McShane, 2019).

## 3 RESULTS

Table 1 presents subjects’ characteristics. Most subjects were at NYHA class II. The body mass index (BMI) values ranged from a classification of eutrophic to obesity grade I. As expected, all fasting glucose and spirometric data were within normal values. Left ventricular ejection fraction ranged from 20.0% to 37.8%. Carotid chemosensitivity was above normal values in only one subject. All subjects were taking beta blockers, drugs targeting the renin‐angiotensin system (ACE inhibitor or AT1 blocker) and diuretics, and just one was taking a vasodilator.

**Table 1:** Subjects’ characteristics

Variable	Mean ± *SD*	Min–Max
Sex	7M/3W	
NYHA (II/III)	6/4	
Age (years)	56 ± 7	44–67
Weight (kg)	78.3 ± 15.6	57–114
BMI (kg/m^2^)	28.0 ± 4.3	20.4–34.4
FGlu (mg/dl)	102.7 ± 10.0	97–122
Creatinine (mg/dl)	0.95 ± 0.16	0.7–1.3
FEV_1_ (%predicted)	78.9 ± 7.1	66.3–89.3
FEV_1_/FVC (%)	101.9 ± 7.9	84.6–116
LVEF Simpson (%)	31.0 ± 6.1	18.5–37.8
Chemo. Sens. (L/min/SpO_2_%)	0.35 ± 0.23	0.15–0.9
Peak workload (W)	62 ± 36	30–145
Peak SAP (mmHg)	162.6 ± 35.9	122–212
Peak DAP (mmHg)	91.6 ± 15.3	70–120
Drugs		
Beta blocker	10	
ACE/AT1	10	
Spironolactone	10	
Furosemide	2	
Hidralazine	1	
Nitrate	1	

Note

*n* = 10 for all variables, with the exception of spirometry variables (*n* = 9) and carotid chemosensitivity (*n* = 9).

Abbreviations: ACE, inhibition of angiotensin‐converting enzyme; AT1, blocker of angiotensin type 1 receptors; BMI, body mass index; Chemo. Sens., carotid chemosensitivity; DAP, diastolic arterial pressure; FEV_1_, forced expiratory volume in 1 s; FGlu, fasting glucose; FVC, forced vital capacity; LVEF, left ventricular ejection fraction; NYHA, New York Heart Association; SAP, systolic arterial pressure.

**Figure 2:**
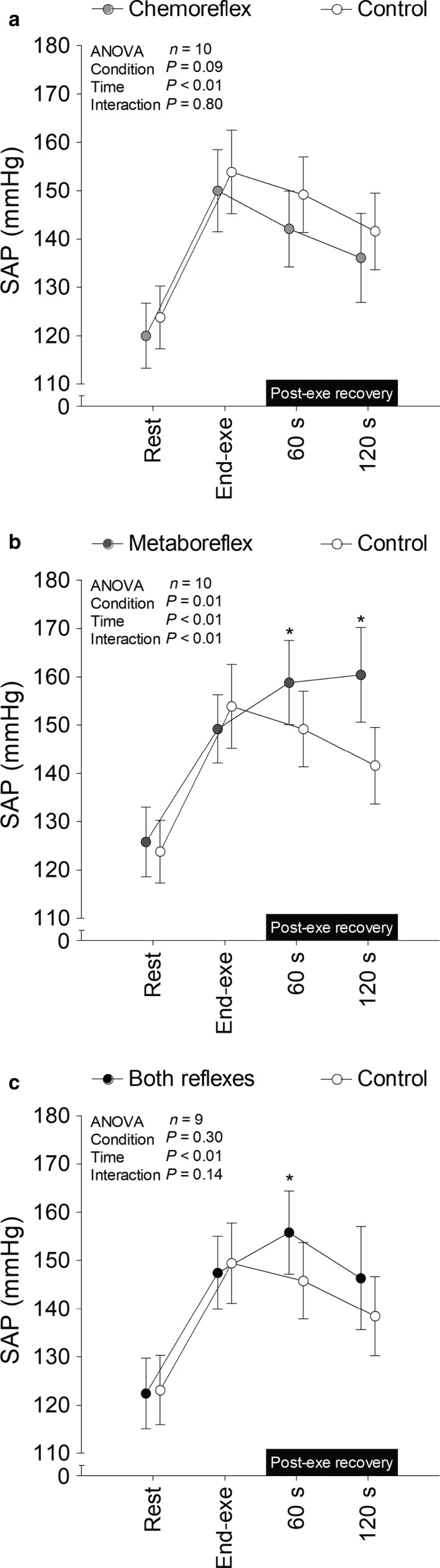
Systolic arterial pressure (SAP) at rest, end‐exercise and during recovery under tonic carotid chemoreflex activity (panel a), muscle metaboreflex activation (panel b) and both reflexes active (panel c) in contrast to both reflexes inactive (i.e., control). *n* = 10 for panels a and b, *n* = 9 for panel c. Data reported as mean ± *SEM* and analyzed by two‐way repeated‐measures ANOVA followed by Fisher´s post hoc. *p < .05 versus control

**Figure 3:**
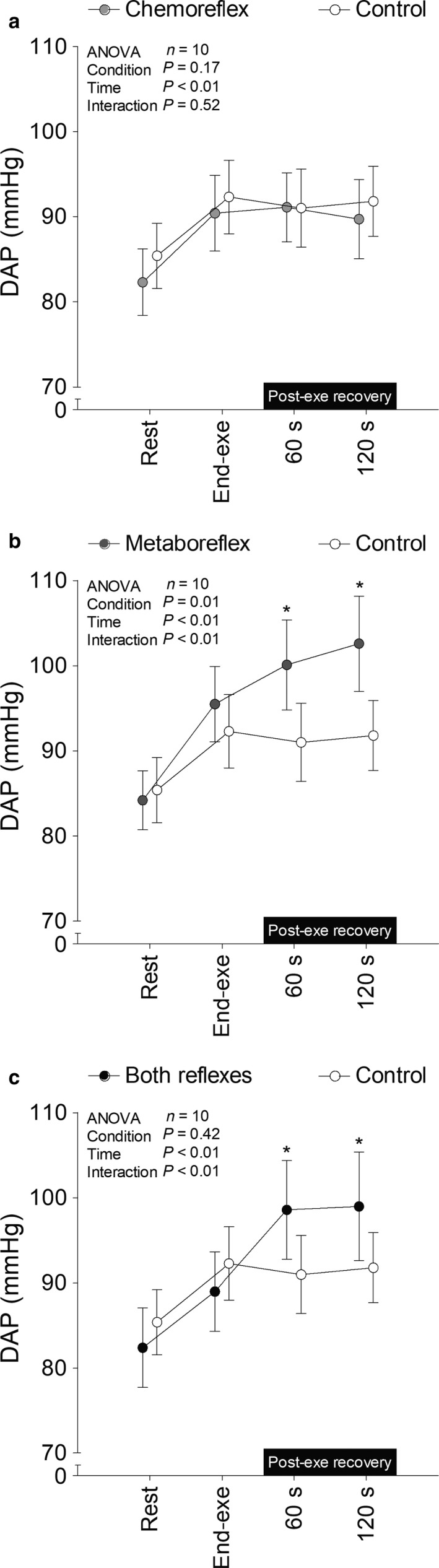
Diastolic arterial pressure (DAP) at rest, end‐exercise and during recovery under tonic carotid chemoreflex activity (panel a), muscle metaboreflex activation (panel b) and both reflexes active (panel c) in contrast to both reflexes inactive (i.e., control). *n* = 10 for panels a, b and c. Data reported as mean ± *SEM* and analyzed by two‐way repeated‐measures ANOVA followed by Fisher's post hoc. *, *p* < .05 versus control

**Figure 4:**
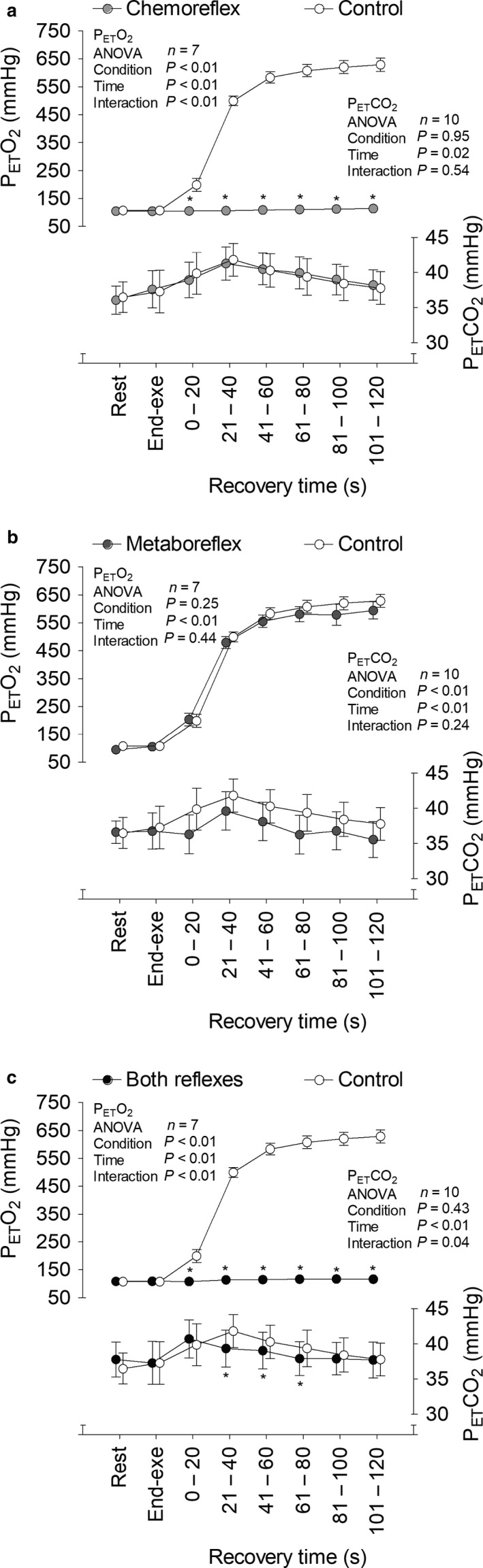
End tidal partial pressure of O_2_ (P_ET_O_2_) and CO_2_ (P_ET_CO_2_) at rest, end‐exercise and during recovery under tonic carotid chemoreflex activity (panel a), muscle metaboreflex activation (panel b) and both reflexes active (panel c) in contrast to both reflexes inactive (i.e., control). *n* = 7 for all P_ET_O_2_ time points, *n* = 7 for resting P_ET_CO_2_ and *n* = 10 other P_ET_CO_2_ time points. In order to raise the statistical power, resting data were not included in the ANOVA analysis. Data reported as mean ± *SEM* and analyzed by two‐way repeated‐measures ANOVA followed by Fisher´s post hoc. *p < .05 versus control

**Figure 5:**
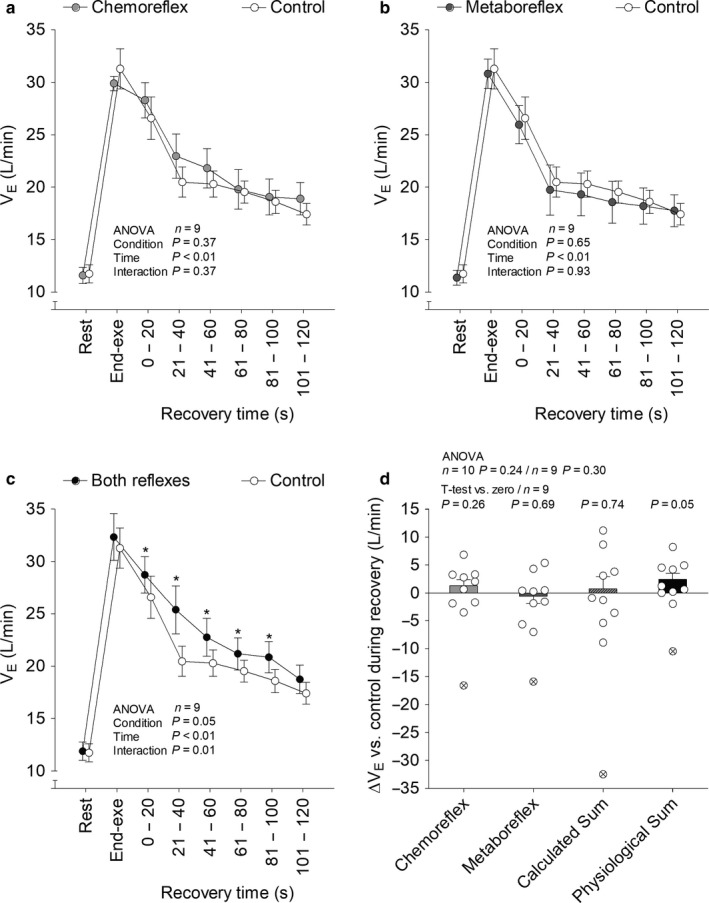
Minute ventilation (${\dot{V}}_{E}$) at rest, end‐exercise and during recovery under tonic carotid chemoreflex activity (panel a), muscle metaboreflex activation (panel b) and both reflexes active (panel c) in contrast to both reflexes inactive (i.e., control). Data reported as mean ± *SEM* and analyzed by two‐way (panels a, b and c; *n* = 9, the outlier was removed), one‐way [panel d; *n* = 10 (with the outlier) or 9 (without the outlier)] repeated‐measures ANOVA (followed by Fisher´s post hoc, if needed) and single mean Student's *t*‐test (panel d; *n* = 9, the outlier was removed). *p < .05 versus control

**Figure 6:**
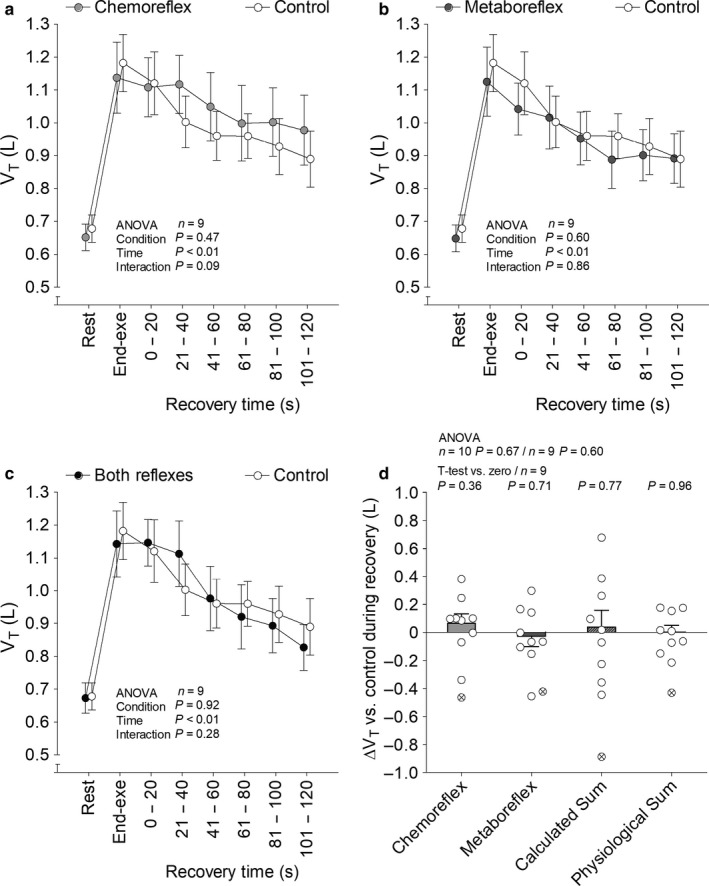
Tidal volume (V_T_) at rest, end‐exercise and during recovery under tonic carotid chemoreflex activity (panel a), muscle metaboreflex activation (panel b) and both reflexes active (panel c) in contrast to both reflexes inactive (i.e., control). Bars represent change versus control (panel d) and crossed out circle represents an outlier. Data reported as mean ± *SEM* and analyzed by two‐way (panels a, b and c; *n* = 9, the outlier was removed), one‐way [panel d; *n* = 10 (with the outlier) or 9 (without the outlier)] repeated‐measures ANOVA and single mean Student's *t*‐test (panel d; *n* = 9, the outlier was removed)

**Figure 7:**
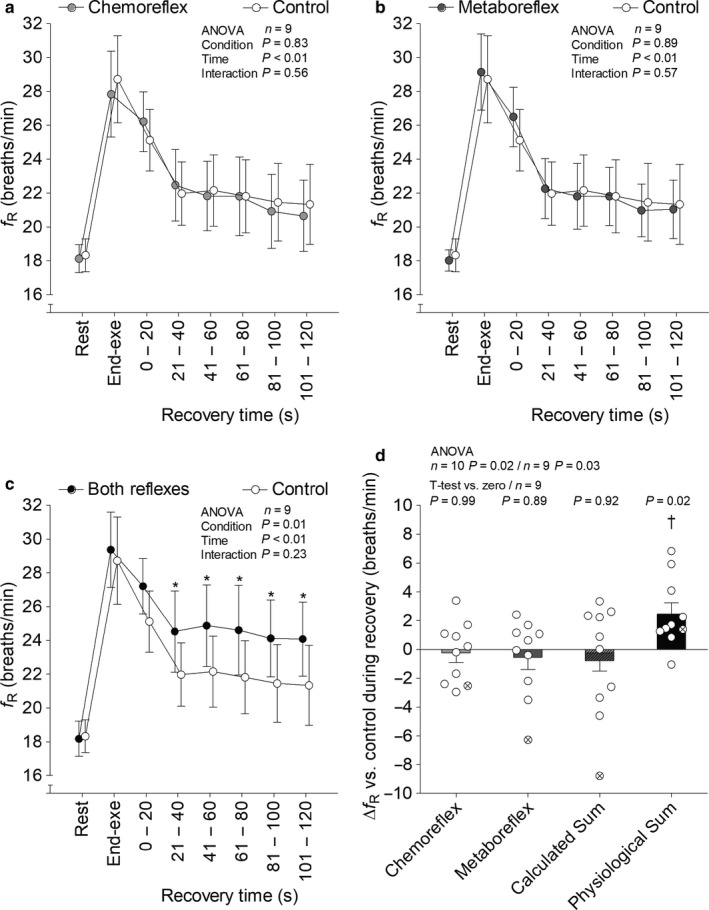
Respiratory frequency (*f*_R_) at rest, end‐exercise and during recovery under tonic carotid chemoreflex activity (panel a), muscle metaboreflex activation (panel b) and both reflexes active (panel c) in contrast to both reflexes inactive (i.e., control). Bars represent change versus control (panel d). Data reported as mean ± *SEM* and analyzed by two‐way (panels a, b and c; *n* = 9, the outlier was removed), one‐way [panel d; *n* = 10 (with the outlier) or 9 (without the outlier)] repeated‐measures ANOVA (followed by Fisher´s post hoc, if needed) and single mean Student's *t*‐test (panel d; *n* = 9, the outlier was removed). *p < .05 versus control. ^†^ *p* < .05 versus all other conditions

SAP (Figure 2), diastolic arterial pressure (DAP; Figure 3), end tidal partial pressure of O_2_ (P_ET_O_2_; Figure 4), P_ET_CO_2_ (Figure 4), ${\dot{V}}_{E}$ (Figure 5), V_T_ (Figure 6), *f*_R_ (Figure 7) were similar between each experimental condition and control at rest and at the end of exercise. SAP and DAP were greater during recovery with muscle metaboreflex activation and both reflexes active as compared to recovery with tonic carotid chemoreflex activity and control. P_ET_O_2_ surpassed 350 mmHg after 21 s of recovery under hyperoxia in all subjects. P_ET_CO_2_ was similar throughout time between tonic carotid chemoreflex activity and control, but it was lower during recovery with muscle metaboreflex activation and both reflexes active versus control.

Recovery with tonic carotid chemoreflex activity or muscle metaboreflex activation did not change ${\dot{V}}_{E}$ and respiratory pattern versus control. V_T_ remained unchanged from control when both reflexes were simultaneously active. On the other hand, ${\dot{V}}_{E}$ and *f*_R_ increased when both reflexes were simultaneously active versus control. Experimental sum and calculated sum were similar for ${\dot{V}}_{E}$, but only experimental sum raised ${\dot{V}}_{E}$ above control (i.e., change greater than zero). Furthermore, experimental sum was greater than calculated sum for *f*_R_. At last, ${\dot{V}}_{E}$ experimental sum neither correlated with ${\dot{V}}_{E}$ separated reflexes effects (carotid chemoreflex: *n* = 9, *r* = .10, *p* = .80; muscle metaboreflex: *n* = 9, *r* = .06, *p* = .89) nor with resting carotid chemosensitivity (*n* = 8, *r* = −.49, *p* = .22). Similarly, *f*_R_ experimental sum neither correlated with *f*_R_ separated reflexes effects (carotid chemoreflex: *n* = 10, *r* = −.29, *p* = .41; muscle metaboreflex: *n* = 10, *r* = .47, *p* = .18) nor with resting carotid chemosensitivity (*n* = 9, *r* = −.42, *p* = .26).

## 4 DISCUSSION

The main results of the present study were that sum of separated reflexes effects did not change ${\dot{V}}_{E}$ and respiratory pattern versus control. On the other hand, ${\dot{V}}_{E}$ and *f*_R_ increased versus control when both reflexes were simultaneously active (experimental sum). Collectively, these results support an interaction between the carotid chemoreflex and the muscle metaboreflex for the regulation of ${\dot{V}}_{E}$ and respiratory pattern. Another noteworthy finding was that ${\dot{V}}_{E}$ and *f*_R_ experimental sums were neither associated with ${\dot{V}}_{E}$ and *f*_R_ separated reflexes effects nor with resting carotid chemosensitivity. The lack of association indicates that the effect mediated by separated reflexes did not predict the effect yielded when both reflexes were simultaneously active. Hence, our results suggest that reflexes interaction should be further explored, particularly in patients who present with altered ${\dot{V}}_{E}$ and respiratory pattern response to exercise, such as those with HFrEF.

### 4.1 Carotid chemoreflex

We found delivery of hyperoxia at the onset of inactive recovery from exercise did not change ${\dot{V}}_{E}$, V_T_ and *f*_R_ versus normoxia when the leg circulation was unobstructed. It is unlikely that the lack of hyperoxia effect was attributed to insufficient inhibition of carotid chemoreceptors. The reason is that data collected in anesthetized cats indicate that 350 mmHg of O_2_ pressure in the arterial blood (PaO_2_) provokes a large reduction in the carotid chemoreceptors activity (Eyzaguirre & Lewin, 1961). We used P_ET_O_2_ as a surrogate of PaO_2_. By means of 100% O_2_ in the inspired air, we reached the 350 mmHg level after 21 s of recovery. Therefore, from this moment on hyperoxia probably induced robust carotid chemoreceptors inhibition. Of note, it is unlikely that aortic chemoreceptors or any other O_2_ sensitive mechanism play a relevant role for the ventilatory control in humans. The reason is that carotid bodies removal in humans, for the sake of asthma treatment in the 1960s (Lugliani, Whipp, Seard, & Wasserman, 1971) or removal of tumors in the neck region (Timmers, Wieling, Karemaker, & Lenders, 2003), almost abolishes the ventilatory response to hypoxia.

A study in patients with right‐sided heart failure induced by pulmonary arterial hypertension employed a similar methodological approach to ours (Paula‐Ribeiro et al., 2019). One hundred percent O_2_ was given in the inspired air 10 s before the onset of active recovery from incremental exercise. Hyperoxia decreased ${\dot{V}}_{E}$ during the first 30 s of active recovery versus normoxia, but then from this time up to 300 s it had no effect. Another study, conducted in healthy humans, showed that the ${\dot{V}}_{E}$ response to transient hypoxia at rest did not vary from pre‐ to post‐heavy‐intensity exercise (Clement et al., 1996), indicating therefore that the carotid chemoreflex sensitivity was unchanged. Together, data from our and previous studies (Clement et al., 1996; Paula‐Ribeiro et al., 2019) support that carotid chemoreceptors, per se, do not seem to contribute at all or contribute to a small part of the ${\dot{V}}_{E}$ recovery from exercise.

### 4.2 Muscle metaboreflex

Obstruction of lower limbs’ circulation maintained arterial pressure at a level superior to the end exercise level, whereas arterial pressure decreased during recovery from exercise without circulatory occlusion. The arterial pressure data thus corroborate previous findings (Alam & Smirk, 1937; Olson, Joyner, & Johnson, 2010; Scott et al., 2002) and indicate that the muscle metaboreflex was operating during recovery with circulatory occlusion. Even though the muscle metaboreflex activity had a clear effect on the arterial pressure, it did not change ${\dot{V}}_{E}$ and respiratory pattern versus control. The absence of an effect on ${\dot{V}}_{E}$ and respiratory pattern has also been reported by other studies in patients with HFrEF (Olson et al., 2010; Scott et al., 2002, 2000), which further supports that muscle metaboreflex activation via circulatory occlusion of both lower limbs during recovery from exercise is ineffective for the ${\dot{V}}_{E}$ and respiratory pattern regulation.

The use of postexercise circulatory occlusion allows maintenance of muscle metaboreflex activity apart from other reflexes activated during exercise like central command (Goodwin, McCloskey, & Mitchell, 1972), muscle mechanoreflex (Silva et al., 2018) and venous distension (Haouzi, Hill, Lewis, & Kaufman, 1999). However, one disadvantage of the method for the study of ${\dot{V}}_{E}$ and respiratory pattern is that it traps a large amount of CO_2_ in the limbs and consequently P_ET_CO_2_ has been shown to decay by about 5 mmHg in patients with HFrEF (Olson et al., 2010), which may reduce the tonic activity of carotid and central chemoreceptors (Forster et al., 2012; Olson et al., 2010). Thus, we used a rebreathing system in an attempt to avoid this problem (Banzett et al., 2000). The CO_2_ control was not perfect though, as P_ET_CO_2_ was about 2 mmHg lower throughout the experiment to activate the muscle metaboreflex and at some time points during the experiment with the presence of both reflexes versus control. However, raising the P_ET_CO_2_ up to 3 mmHg did not change ${\dot{V}}_{E}$ during postexercise muscle metaboreflex activation in healthy young men (Alghaith, Balanos, Eves, & White, 2019). Thus, it is unlikely that P_ET_CO_2_ was an issue for our data interpretation.

### 4.3 Interaction between reflexes

Neither active carotid chemoreflex nor active muscle metaboreflex in separation changed ${\dot{V}}_{E}$, V_T_ and *f*_R_ versus the control condition. Sum of separated reflexes effects remained similar to control. On the other hand, ${\dot{V}}_{E}$ and *f*_R_ increased above the control condition level when both reflexes were simultaneously active. Even using the single mean Student's *t*‐test, which is a less strict analysis, separated reflexes and calculated sum remained unchanged from control. Therefore, the absence of separated reflex effects and calculated sum effect does not seem to be attributed to lack of statistical power. Unexpectedly, interaction between the carotid chemoreflex and the muscle metaboreflex yielded an additive effect (i.e., experimental sum similar to calculated sum) on the ${\dot{V}}_{E}$ and a hyperadditive effect on the *f*_R_ (i.e., experimental sum greater than calculated sum). Breathing with a facemask could have some effect on ${\dot{V}}_{E}$ and breathing pattern (Bloch, Barandun, & Sackner, 1995; Sackner, Nixon, Davis, Atkins, & Sackner, 1980), but the effect would be systemically present during all experiments which makes the use of a facemask an unlikely explanation for these results. It is also unlikely that P_ET_CO_2_ was an issue, as mentioned earlier. An alternative explanation could be the level of carotid chemosensitivity in our subjects. Just one subject presented resting carotid chemosensitivity higher than the superior range of normal (Chua et al., 1997). Thus, perhaps the effect of simultaneous reflexes participation would be greater in patients with higher carotid chemoreflex activity and sensitivity. Of note, during exercise, the carotid chemoreflex and the muscle metaboreflex possibly interact with other operating reflexes like central command (Goodwin et al., 1972), muscle mechanoreflex (Silva et al., 2018) and venous distension (Haouzi et al., 1999). As a result, the effect herein observed could have been greater if we had assessed an interaction between the carotid chemoreflex and the muscle metaboreflex during exercise, rather than recovery (Lam et al., 2019).

We did not use hypoxia to stimulate the carotid chemoreceptors in present study for the sake of patients’ safety. Thus, our data reflects the effect of tonic carotid chemoreceptors activity, rather than its sensitivity to hypoxia. Importantly, patients with HFrEF infrequently develop hypoxemia during exercise (Smith & Olson, 2019; Van Iterson et al., 2017), and so the assessment of tonic carotid chemoreceptors regulation of exercise hyperpnea enriches the external validity of our results. Some studies in healthy young adults have employed hypoxia during concomitant activation of skeletal muscle afferents. For instance, simultaneous carotid chemoreceptors activation via hypoxia and muscle metaboreflex activation via posthandgrip ischemia tended (*p* = .14) to provoke greater ${\dot{V}}_{E}$ response than the sum of ${\dot{V}}_{E}$ responses to each separated stimulus in healthy young men, in spite of P_ET_CO_2_ being ~7 mmHg lower during hypoxic posthandgrip ischemia versus normoxic posthandgrip ischemia (Edgell & Stickland, 2014). Consequently, the ${\dot{V}}_{E}$ difference between experimental and calculated sums could have been clearer at comparable P_ET_CO_2_ level. Another study showed that simultaneous carotid chemoreceptors activation, via hypoxia, and muscle mechanoreflex activation, via passive limb movement, under isocapnia provoked a hyperadditive ${\dot{V}}_{E}$ response in healthy young adults (Silva et al., 2018). Therefore, use of hypoxia in healthy young humans to increase the carotid chemoreceptors activity supports the aforementioned hypothesis that the ${\dot{V}}_{E}$ effect reported in the present study could have been greater in patients with higher carotid chemoreflex activity and sensitivity.

Our study was not designed to dissect where and how the interaction between the carotid chemoreflex and the muscle metaboreflex takes place, but it is conceivable that these reflexes interacted both at the central nervous system and at the periphery. Afferents from carotid chemoreceptors (Accorsi‐Mendonça et al., 2011) and muscle metaboreceptors synapse at close regions in the nucleus of the tractus solitarius (Potts et al., 2003). Thus, afferent signals could be potentiated at this place, similar to what has been described for the interaction between afferent signals from arterial baroreceptors and muscle mechano‐ and metabo‐receptors (Potts et al., 2003). Furthermore, activation of the muscle metaboreflex could increase carotid chemoreceptors activity via increased sympathetic nerve activity to carotid bodies (O'regan, 1981). The increased sympathetic activity, in turn, could change carotid chemoreceptors activity either directly (Eyzaguirre & Lewin, 1961) or indirectly via alpha receptors‐mediated vasoconstriction of arterioles that perfuse carotid chemoreceptors (Almaraz, Perez‐Garcia, Gomez‐Nino, & Gonzalez, 1997). It is important to mention that these hypotheses are based on animal model studies, and so, their translation to patients with HFrEF is limited.

### 4.4 Implications

Our results support that the interaction between the carotid chemoreflex and the muscle metaboreflex raises ${\dot{V}}_{E}$ in a *f*_R_‐dependent manner. The *f*_R_ difference between experimental and calculated sums was of 2 cycles/min. At a first glance it may appear to be an effect of unimportant magnitude. However, evidence indicates that at similar level of ${\dot{V}}_{E}$ (~45 L/min), patients with HFrEF showed *f*_R_ 7 cycles/min greater than healthy controls (Smith & Olson, 2019). V_T_ was similar to controls (Smith & Olson, 2019). As a result, the increased *f*_R_ provoked hypocapnia (Smith & Olson, 2019). Estimation based on alveolar ventilation and $\dot{V}$CO_2_ data indicates that the hypocapnia could have been eliminated if the excessive *f*_R_ response decreased by 4 cycles/min. Thus, if the interaction between the carotid chemoreflex and muscle metaboreflex contributes to an extra *f*_R_ increase of 2 cycles/min during exercise, it would represent 50% of the exaggerated *f*_R_ response to exercise at an iso‐${\dot{V}}_{E}$ level.

The consequences of *f*_R_‐mediated exercise hyperventilation in patients with HFrEF may include increased dead space ${\dot{V}}_{E}$ and increased work of breathing (Smith & Olson, 2019), which in combination can accentuate the perception of dyspnea (Andreas, Vonhof, Kreuzer, & Figulla, 1995; Morosin et al., 2018). The ensuing augmented recruitment of respiratory muscles may induce competition for distribution of the patients’ low cardiac output between respiratory and locomotor muscles (Borghi‐Silva et al., 2008), likely adding to restrain locomotor muscles perfusion (Borghi‐Silva et al., 2008). As a result, lower limb discomfort may increase and, along with the aforementioned dyspnea, can contribute to reduce the exercise tolerance (Borghi‐Silva et al., 2008). Therefore, remains to be investigated whether interaction between the carotid chemoreflex and the muscle metaboreflex contributes to respiratory, cardiovascular and perceptual responses to exercise. If it does contribute, attenuation of the interaction between the carotid chemoreflex and the muscle metaboreflex could be beneficial for patients with HFrEF, which could probably be achieved, for example, via exercise training (Negrao, Middlekauff, Gomes‐Santos, & Antunes‐Correa, 2015).

## 5 CONCLUSION

The carotid chemoreflex and the muscle metaboreflex interacted to ${\dot{V}}_{E}$ regulation in a *f*_R_‐dependent manner in patients with HFrEF. Therefore, if this interaction operates during exercise, it can have some contribution to the HFrEF exercise hyperventilation.

## AUTHORS’ CONTRIBUTION

ACM, LCV, JACT, MLR, HV, ACLN, and BMS designed the project; ACM, EACG, JACT, MLR, and HV collected the data; ACM, JACT, MLR, and BMS analyzed the data; ACM and BMS involved in statistical analyses; ACM, LCV, EACG, JACT, MLR, HV, ACLN, and BMS interpreted the data; ACM and BMS drafted the manuscript; and ACM, LCV, EACG, JACT, MLR, HV, ACLN, and BMS revised the final manuscript.
